# Compact Design of a 50° Field of View Collimating Lens for Lightguide-Based Augmented Reality Glasses

**DOI:** 10.3390/mi16111234

**Published:** 2025-10-30

**Authors:** Wen-Shing Sun, Yi-Lun Su, Ying-Shun Hsu, Chuen-Lin Tien, Nai-Jen Cheng, Ching-Cherng Sun

**Affiliations:** 1 Department of Optics and Photonics, National Central University, Chungli 32001, Taiwan; wssun@dop.ncu.edu.tw (W.-S.S.); a0357841@gmail.com (Y.-L.S.); dmcandymurray@yahoo.com.tw (Y.-S.H.); ccsun65298@gmail.com (C.-C.S.); 2 Department of Electrical Engineering, Feng Chia University, Taichung 40724, Taiwan; 3 Institute of Photonics Engineering, National Kaohsiung University of Science and Technology, Kaohsiung 807618, Taiwan; njcheng@nkust.edu.tw

**Keywords:** compact collimating lens, augmented reality, microdisplay, modulation transfer function

## Abstract

Designing a compact collimating lens system for augmented reality (AR) applications presents significant optical challenges. This paper presents a compact, 50-degree field-of-view collimating lens system explicitly designed for lightguide-based AR glasses. The compact collimating lens is designed for a 0.32-inch microdisplay and consists of four plastic aspherical lenses. The optical design results in a collimating lens with a F-number of 2.17 and an entrance pupil diameter of 4 mm. Optical distortion is less than 0.29%, and the modulation transfer function (MTF) is greater than 0.23 at 250 cycles/mm. The overall lens diameter, including the lens barrel, measures 10.16 mm, while the lens length is 11.48 mm. The lens volume is 0.93 cm^3^, and its mass is 1.08 g. Compared to existing collimator designs, this approach significantly improves the trade-off between field of view, optical quality, and device miniaturization. The proposed design supports integration with 0.32-inch microdisplays, making it a practical and manufacturable solution for next-generation AR eyewear. This paper presents innovative contributions to the optical design of AR glasses, demonstrating considerable potential in reducing size and weight, and optimizing optical performance.

## 1. Introduction

In recent years, augmented reality (AR) lightguide displays have gained increasing attention due to their potential to deliver lightweight, wide field-of-view (FOV), and high-quality visual experiences in wearable systems. Among the various optical coupling and image-forming technologies, volume holographic optical elements (VHOEs) have emerged as a promising solution for achieving high diffraction efficiency, precise wavefront control, and compact system integration [1,2,3,4]. The collimating lens is a crucial optical component of head-mounted displays (HMDs), responsible for projecting images from the display to the human eye. Extensive research has been devoted to enhancing the design, simulation, and fabrication of VHOE-based lightguides. For example, Tsai [5] integrates Kogelnik’s coupled-wave theory with ray tracing modeling to optimize lightguide combiners, improving efficiency, reducing module volume, and enhancing image quality. Similarly, Lee [6] embeds coupled-wave theory into ray-tracing-based simulations, enabling accurate modeling of VHOE diffraction and image performance in AR systems. Efforts toward structural optimization have also been reported.

The resolution of the collimating lens can significantly affect the overall optical quality of the system, including light source utilization, illumination uniformity, color reproduction, and image performance. Hua [7] present the design of an ultralight and compact collimating lens for a HMDs, utilizing a 1.35-inch microdisplay with a resolution of 640 × 480 pixels, providing a 52.4 degrees FOV, an F-number of 2.92, an entrance pupil diameter of 12 mm, and an effective focal length of 35 mm, with a total volume of 3.53 cm^3^. In 2007, Hua [8] proposed a polarized head-mounted projection display (p-HMPD) and developed a compact optical system using a 1.3-inch microdisplay of 640 × 480 pixels, achieving 56 degrees FOV, an F-number of 3.2, an entrance pupil diameter of 10 mm, and an effective focal length of 31.5 mm, with a reduced lens volume of 3.15 cm^3^. In 2008, Zhang and Hua [9] reported the design of a p-HMPD prototype employing a pair of high-resolution ferroelectric liquid-crystal-on-silicon (FLCOS) microdisplays, offering higher optical efficiency. The light engine used a 0.88-inch microdisplay at 1280 × 1024 pixels, providing a 55-degree FOV, an F-number of 2.16, an entrance pupil diameter of 10 mm, and an effective focal length of 21.6 mm, with a compact lens volume of approximately 2.66 cm^3^. Pan [10] designed a collimating lens for a geometric waveguide display using a 0.62-inch microdisplay with 800 × 600 pixels, achieving a 30-degree FOV, an F-number of 3.66, an entrance pupil diameter of 8 mm, and an effective focal length of 29.3 mm, with a total lens volume of 12.45 cm^3^. Tsai [11] proposed an optical see-through head-mounted display (OST-HMD) system in which a rotationally symmetric eyepiece with a wide 60-degree FOV was coupled with a dihedral corner reflector array (DCRA). The system employed a 0.7-inch microdisplay with a resolution of 1920 × 1080 pixels, an F-number of 2.58, an entrance pupil diameter of 6 mm, an effective focal length of 15.46 mm, and a lens volume of 9.76 cm^3^. Wu [12] proposes a curved variable-period grating to replace conventional in-coupler and collimating lens, achieving about 39.3% thickness reduction, 70% system efficiency, and a 36.6 degrees FOV. Weng [13] proposed a waveguide display scheme based on the coupling-collimating system. This system incorporated a freeform liquid crystal diffractive optical element within the waveguide and a freeform element outside the waveguide, achieving a FOV of 35 degrees, an F-number of 2.5, an entrance pupil diameter of 9 mm, and an effective focal length of 22.5 mm.

Sun [14] designed a collimating lens with a 30-degree FOV for a 0.35-inch microdisplay with 1280 × 720 pixels. The entrance pupil diameter of the lens is 14 mm, an effective focal length of 16.443 mm with an F-number of 1.175, and the lens volume is 25.02 cm^3^. In 2025, Sun [15] proposed a wide FOV of a 65-degree collimating lens design. The entrance pupil diameter of this system is 10 mm, the effective focal length is 20.458 mm, and the F-number is 2.046. It features a 1.03-inch microdisplay with a resolution of 2560 × 2560 pixels; the lens volume is 22.61 cm^3^. This paper presents innovative contributions to the optical design of augmented reality glasses, demonstrating considerable potential in reducing size and weight, and optimizing optical performance. The final lens volume is 0.93 cm^3^, and the mass is 1.08 g.

## 2. Optical Design Method

### 2.1. Subsection Microdisplay Specifications

The design of a collimating lens with a 50-degree FOV was a 0.32-inch microdisplay. The specifications are shown in Table 1. In the specifications, the pixel size was 8.1 μm × 8.1 μm. The effective area of 6.48 mm × 4.86 mm was calculated for the diagonal length of 8.1 mm, which could be used to define the paraxial image height of 4.05 mm.

### 2.2. Five Reference Rays on the Entrance Pupil

**Figure 1:**
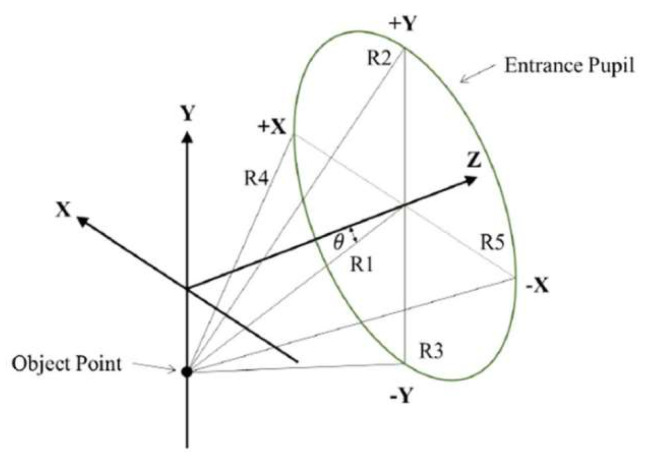
The five reference rays (R1, R2, R3, R4, and R5) defined on the entrance pupil.

In the CODE V optical design software, each field of view is associated with five reference rays, denoted as R1, R2, R3, R4, and R5, as illustrated in Figure 1. Among them, R1 represents the chief ray, which passes through the center of the entrance pupil and forms an angle θ with the optical axis, corresponding to the half FOV. The remaining rays—R2, R3, R4, and R5—are marginal rays that pass through the top edge (+Y), bottom edge (−Y), left edge (+X), and right edge (−X) of the entrance pupil, respectively.

### 2.3. Clear Aperture Calculation of the Lens

**Figure 2:**
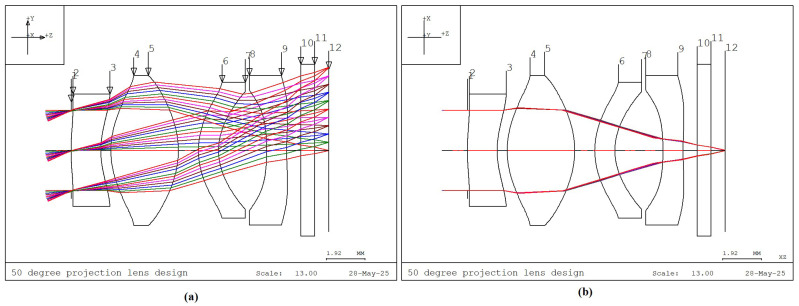
Ray trace diagrams of the 50° FOV collimating lens design. (**a**) Lens elements and reference rays in the Y–Z plane. (**b**) Lens elements and reference rays in the X–Z plane.

The maximum half FOV of 25° is defined as the full field (1.0 field) to define the clear aperture of the collimating lens system. To ensure uniform image quality, the 1.0 field is divided into 11 evenly spaced points for optimization, corresponding to 0.0, 0.1, 0.2, …, up to 1.0 field. In CODE V, the clear aperture of each lens surface is calculated by performing real ray tracing of five reference rays (R1 to R5) per field point, resulting in a total of 55 rays. The maximum footprint of these rays on each surface defines the required clear aperture of that surface. The collimating lens system in this study covers a FOV of 50°. It comprises four lens elements and one protective window, as illustrated in Figure 2. The optical axis is aligned with the Z-axis. Figure 2a shows the Y–Z cross-sectional view of the lens system and the ray traces for reference rays R1, R2, and R3 across all fields (a total of 33 rays). Figure 2b shows the X–Z plane, which includes reference rays R4 and R5 (a total of 22 rays). Hence, to fully determine the clear aperture of each lens surface, ray tracing of all 55 reference rays is required.

### 2.4. Relationship Among Half FOV, Image Height, Entrance Pupil Diameter, and Effective Focal Length

**Figure 3:**
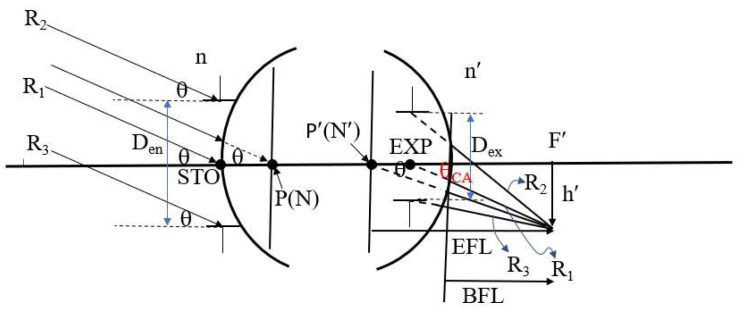
Geometric relationship among half field angle, image height, effective focal length, and entrance pupil diameter.

Figure 3 illustrates the relationships among half FOV, image height, effective focal length (EFL), and entrance pupil diameter. Points P and P′ denote the first and second principal points, respectively, while N and N′ denote the first and second nodal points. The symbols n and n′ represent the refractive indices of the object and image spaces, respectively. F′ denotes the rear focal point, h′ represents the image height, and STO indicates the position of the aperture stop. In this system, the aperture stop is located on the first surface of the lens, causing the entrance pupil to coincide with the aperture stop. EXP indicates the exit pupil position, while D_en_ and D_ex_ represent the diameters of the entrance and exit pupils, respectively. The symbol θ_CA_ refers to the chief ray angle in the image space. EFL is the lens’s effective focal length, and BFL is the back focal length. Assuming that the object space half FOV is denoted by θ, and the object is located at infinity, then the chief ray, marginal ray, and the ray passing through the first principal point (P) will all make the same angle θ. If the object space and image space refractive indices (n and n′) are equal (typically air), then the first principal point P coincides with the first nodal point N, and the second principal point P′ coincides with the second nodal point N′. Due to the nodal point property, the incident angle at N equals the emergent angle at N′, thereby establishing the following relationship between image height h′ and the effective focal length EFL, as shown in Equation (1)

(1) $$\tan\theta =\frac{h^{\prime }}{EFL}.$$

Furthermore, the relationship among the F-number, the entrance pupil diameter (Den), and the effective focal length (EFL) is given by Equation (2).

(2) $$F-number=\frac{EFL}{D_{en}}.$$

The clear aperture of each lens surface decreases as the entrance pupil diameter (D_en_), image height (h′), or half field angle (θ) decreases. Since the half FOV θ is fixed at 25°, Equation (1) implies that a smaller image height h′ corresponds to a shorter effective focal length (EFL). According to Equation (2), if the entrance pupil diameter D_en_ is fixed at 4 mm, a decrease in EFL also results in a lower F-number.

### 2.5. Entrance Pupil Position and Size

**Figure 4:**
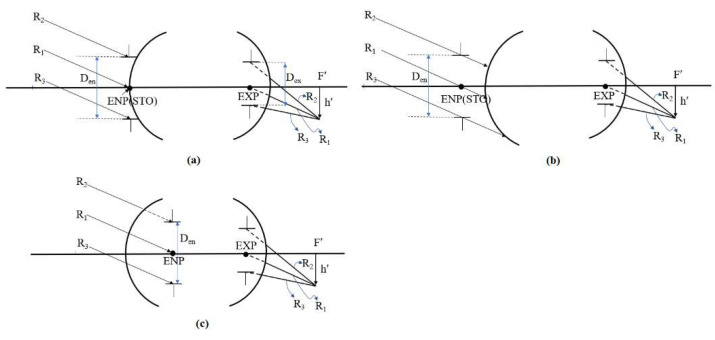
Relationship between the entrance pupil position and the clear aperture of the first lens: (**a**) entrance pupil located on the first surface; (**b**) entrance pupil located in front of the first surface; (**c**) entrance pupil located behind the first surface.

The aperture stop location and size determine the minimum clear aperture of a lens system. When the aperture stop is placed on the first surface or before the lens, the entrance pupil (ENP) coincides with the aperture stop. Its size is identical to that of the aperture stop, and directly determines the clear aperture of the first lens, which in turn influences the clear aperture of the second lens, as illustrated in Figure 4. As shown in Figure 4a, with the aperture stop on the first surface of lens, the minimum clear aperture occurs on the first lens. Figure 4b shows the aperture stop before the lens, where the entrance pupil coincides with the aperture stop. The ray height of reference ray R3 on the first surface of the lens is larger than the entrance pupil radius. If the aperture stop position moves toward the object space, the farther the distance the aperture stop is from the first surface of the lens, the greater the ray height of reference ray R3 on the first surface, and a larger clear aperture of the first lens, and increasing the overall lens volume. Figure 4c shows the entrance pupil behind the first surface, which no longer coincides with the aperture stop. The ray height of reference ray R2 on the first surface of the lens is larger than the entrance pupil radius. If the entrance pupil position moves toward the image space, the farther the entrance pupil is from the first surface, the greater the ray height of reference ray R2 on the first surface, and a larger clear aperture of the first lens, increasing the overall lens volume.

### 2.6. Exit Pupil Position and Size

The FOV 50-degree collimating lens design consists of four lenses. Since the aperture stop is located on the first lens, the first lens has the smallest clear aperture. However, because the third and fourth lenses are farther away from the first surface of the lens, the clear apertures of the third and fourth lenses tend to increase. We must suppress the factors that cause the clear apertures of the third and fourth lenses to become larger.

**Figure 5:**
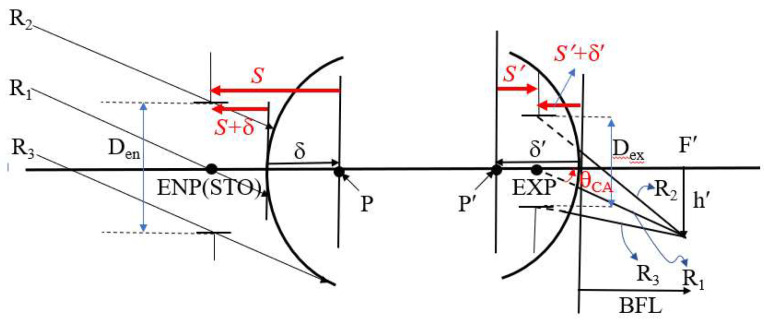
Relationship between the exit pupil position and the clear aperture of the last lens surface.

Figure 5 defines the relationship between the exit pupil position and size and the clear aperture of the rearmost surface of the lens. Distances are directional, with negative values to the left and positive values to the right. S is the distance from the first principal point (P) to the entrance pupil (ENP), and *S*′ is the distance from the second principal point (P′) to the exit pupil (EXP). δ is the distance from the first surface of the lens to the first principal point, and δ′ is the distance from the rearmost surface of the lens to the second principal point. Therefore, the distance from the first surface of the lens to the entrance pupil is *S* + δ, and the distance from the rearmost surface of the lens to the exit pupil is *S*′ + δ′. If the exit pupil is at the second principal point, the image space chief ray angle θ_CA_ equals the half FOV θ, θ_CA_ = 25°. If the exit pupil position is to the right of the second principal point, θ_CA_ > 25°; and if it is to the left of the second principal point, θ_CA_ < 25°. As θ_CA_ decreases, the exit pupil becomes farther from the rearmost surface of the lens (excluding the flat glass), resulting in a larger clear aperture at the rearmost lens. If θ_CA_ = 0°, the exit pupil position is at infinity, meaning the image space chief ray is incident perpendicularly on the image plane (image telecentric), and the clear aperture of the rearmost element is at its maximum. Similarly, as θ_CA_ increases (θ_CA_ > 25°), the exit pupil becomes closer to the rearmost surface of the lens, resulting in a smaller clear aperture. If the exit pupil is at the rearmost surface of the lens, the clear aperture of the rearmost lens is at its minimum, calculated as the exit pupil diameter (D_ex_). Image height h′ and exit pupil diameter are also important factors influencing the clear aperture of the rearmost lens.

The exit pupil distance *S*′ can be calculated from the relationship between S and *S*′ and the EFL of the lens as shown in Equation (3) [16]:

(3) $$\frac{1}{S\prime }=\frac{1}{S}+\frac{1}{EFL}.$$

The image space chief ray angle *θ*_C__A_, is determined according to Equation (4).

(4) $$\theta_{CA}=\tan^{-1}(\frac{h^{\prime }}{BFL-S^{\prime }-\delta^{\prime }}).$$

The exit pupil diameter (D_ex_) is obtained through the relationship between the lens lateral magnification (M_T_) and the entrance pupil diameter (D_en_), as given in Equation (5) [16].

(5) $$M_{T}=\frac{S\prime }{S}=\frac{D_{ex}}{D_{en}}.$$

### 2.7. Relationship Between Effective Focal Length and Angular Magnification

The angular magnification (M_p_) of the magnifying glass [17] can be obtained

(6) $$M_{P}=-\frac{d_{o}}{f^{\prime }}$$

In the design described in this paper, the collimating lens function like a magnifying glass. As a result, the image is magnified after passing through the collimating lens. Let d_o_ be the distance of distinct vision, d_o_ = −250 mm, *f′* denote the image space focal length of the collimating lens, where *f′* = 8.68 mm. Let M_p_ represent the angular magnification. M_p_ = 28.8° The effective area of the microdisplay is 6.48 mm × 4.86 mm, and then the horizontal length and the vertical length of the virtual image when the eyes can see from the collimating lens at a distance of 250 mm can be obtained by 186.624 mm × 139.968 mm [15].

## 3. Design Result

This section may be divided by subheadings. It should provide a concise and precise description of the design results, their interpretation, as well as the design conclusions that can be drawn.

### 3.1. Lens Specifications and Lens Data

The specifications of the collimating lens are shown in Table 2, with the aperture stop location on the first surface. The entrance pupil diameter is 4 mm, and the lens has a diagonal FOV of 50 degrees. A horizontal FOV of 40.94 degrees is required. The F-number is 2.17, the focal length is 8.68 mm, and the image height is 4.05 mm. The lens resolution is set at 62 cycles/mm, which corresponds to an angular resolution requirement of 20 pixels per degree (PPD). The angular magnification of the collimating lens is 28.8, and the design specifications are summarized in Table 2.

**Figure 6:**
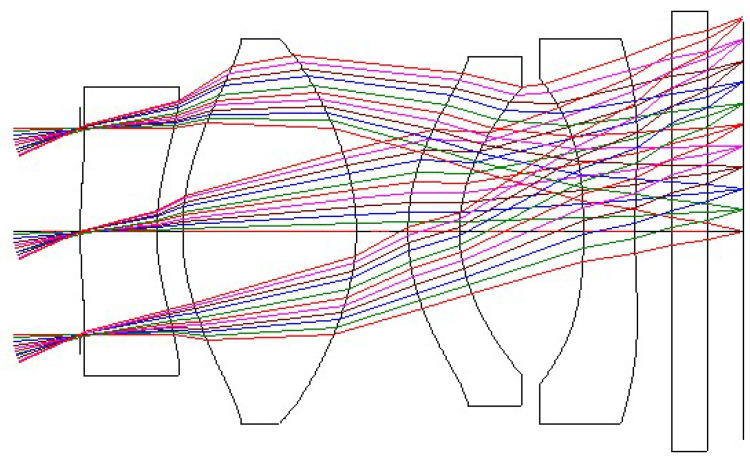
Optical layout of the 50° FOV collimating lens design.

The lens layout of the ultra-thin collimating lens with a FOV of 50° is shown in Figure 6, and the corresponding lens data and aspherical surface data are listed in Table 3 and Table 4, respectively. Surface Number is the order of the surface numbers of the collimating lens. K represents the conic constant. A, B, C, and D represent the coefficients of the 4th, 6th, 8th, and 10th order terms of the aspheric surface. The K value is defined as: K > 0 represents an elliptical surface with the minor axis on the optical axis, K = 0 represents a spherical surface, −1 < K < 0 represents an elliptical surface with the major axis on the optical axis, K = −1 represents a paraboloid, and K < −1 represents a hyperboloid. The system comprises four plastic aspheric lenses and one protective cover glass for the microdisplay.

### 3.2. Analysis of Collimating Lens Image Quality

**Figure 7:**
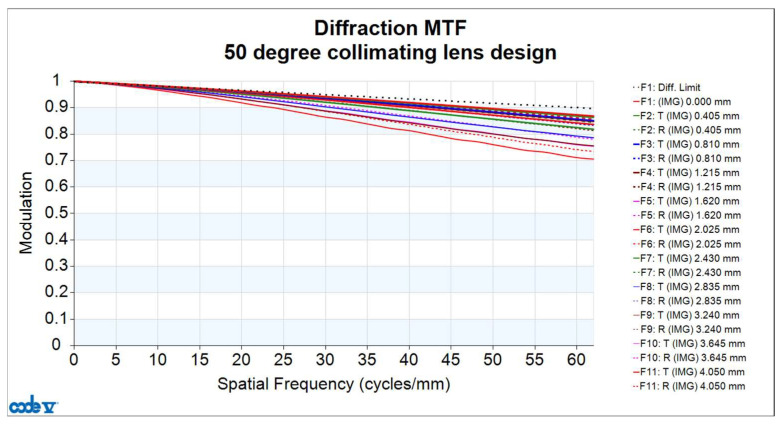
Modulation Transfer Function (MTF) curve of the FOV 50° collimating lens design at a spatial frequency of 62 cycles/mm.

**Figure 8:**
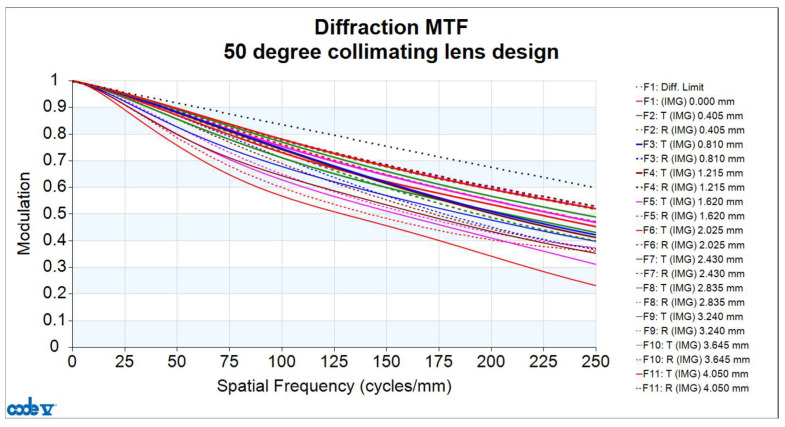
Modulation Transfer Function (MTF) curve of the FOV 50° collimating lens design at a spatial frequency of 250 cycles/mm.

The image quality analysis of the collimating lens includes evaluation of the Modulation Transfer Function (MTF), optical distortion, lateral color, and relative illumination. Figure 7 shows the MTF curves of the lens, where the horizontal axis represents spatial frequency. Given that the pixel size of the microdisplay is 8.1 μm, the Nyquist frequency is determined to be 62 cycles/mm. At this frequency, the MTF value is at least 0.711. Since the resolution limit is constrained by the pixel size of the microdisplay (i.e., 62 cycles/mm), this defines the upper bound of the system performance when the display is considered. However, if the MTF performance of the collimating lens is evaluated independently—disregarding the pixel limitations of the microdisplay—and the same image plane position and image area are assumed, the spatial frequency can be extended up to 250 cycles/mm. At this frequency, the MTF value remains above 0.232, as shown in Figure 8, corresponding to an angular resolution of up to 79 pixels per degree (PPD).

**Figure 9:**
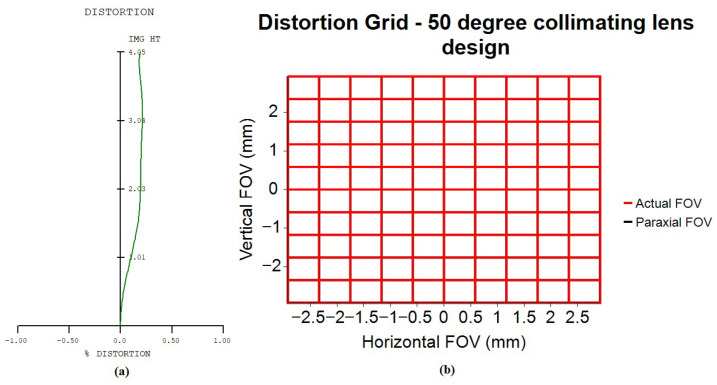
Optical distortion curve for 50-degree FOV collimating lens. (**a**) the optical distortion plot; (**b**) the corresponding distortion grid chart.

Figure 9a shows the optical distortion plot for the collimating lens design. The maximum optical distortion is 0.2128%, occurring at the 0.8 field position. The TV distortion, defined as the absolute difference between the maximum and minimum optical distortion values from the 0.6 to 1.0 field region, is 0.0195%. Figure 9b presents the corresponding distortion grid chart. The horizontal and vertical axes represent the image heights in the horizontal and vertical directions. The black curve illustrates the variation in paraxial image height, while the red curve represents the actual image height. The collimator design in this study exhibits minimal TV distortion (0.0195%), resulting in a straight red curve without distortion. In addition, the collimator design in this article also exhibits minimal optical distortion (0.2128%), allowing the red curve to overlap with the black curve, making the black curve effectively invisible.

**Figure 10:**
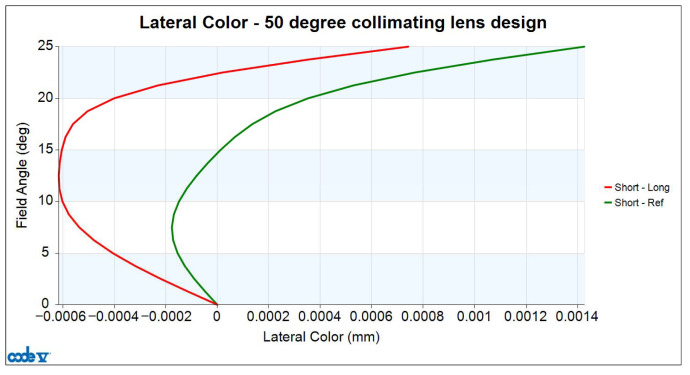
Lateral color curve for 50-degree FOV collimating lens.

Lateral chromatic aberration is defined as the difference in image height on the imaging plane when tracing real light rays of different wavelengths. The lens design presented in this paper is based on three wavelengths: a short wavelength (F-line, 0.4861 µm), a reference wavelength (d-line, 0.5876 µm), and a long wavelength (C-line, 0.6563 µm). The design values for lateral color are shown in Figure 10. In this figure, the horizontal axis represents lateral color in mm, and the vertical axis represents the half-field angle in degrees. The red curve represents the lateral color values for the short and long wavelengths across different field angles. The maximum value of the red curve occurs at a half FOV of 25°, with a value of 0.742 µm. The green curve represents the lateral color values for the short and reference wavelengths. The maximum value of the green curve occurs at a half FOV of 25°, with a value of 1.426 µm. Lateral chromatic aberration must be smaller than the microdisplay pixel size of 8.1 µm; otherwise, the lens will be unable to resolve it, and lateral chromatic aberration will occur easily. The lateral chromatic aberration of the collimator designed in this paper is much smaller than the microdisplay pixel size of 8.1 µm, so the lateral chromatic aberration value can be ignored.

**Figure 11:**
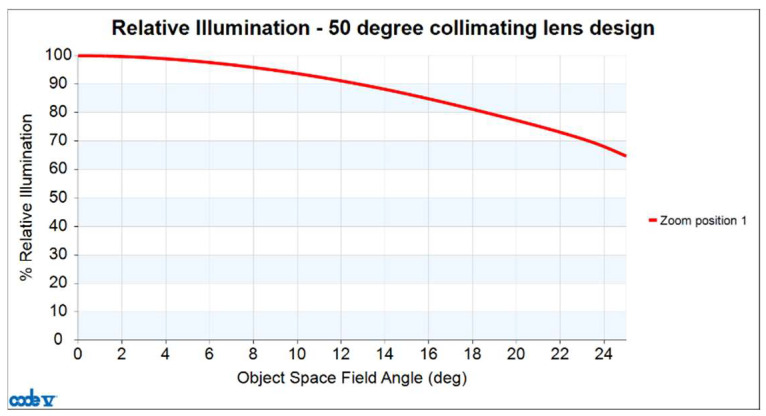
Relative illumination curve for 50-degree FOV collimating lens.

Finally, the relative illumination curve is shown in Figure 11. The red curve shows the relative illumination values at different half-field angles. In this design, the relative illumination is greater than 65%.

### 3.3. Lens Manufacturing and Tolerance Analysis

The tolerances [18] include the radius of curvature (DLF), the cylinder irregularity (CYD, CYN), the thickness (DLT), the refractive index (DLN), the V-number (DLV), the wedge (TRX, TRY), the tilt (BTX, BTY), and the displacement (DSX, DSY). The range of tolerance parameters set for this lens design is shown in Table 5.

**Figure 12:**
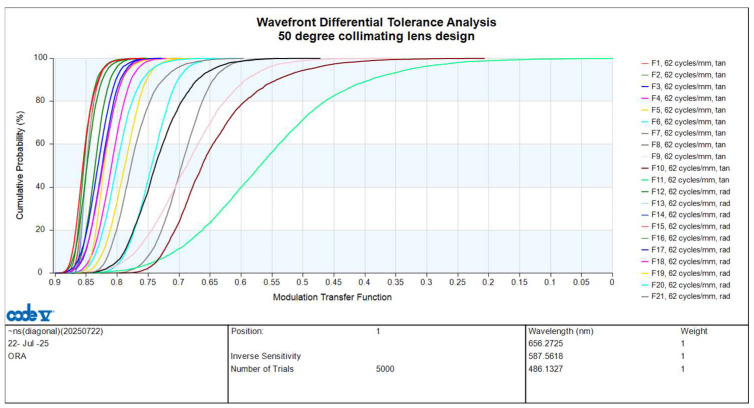
Tolerance analysis curve for 50-degree FOV collimating lens.

The cumulative distribution probability curve for tolerance analysis is found by combining the effects of all tolerance items. The vertical axis represents the cumulative distribution probability, and the horizontal axis represents the MTF. Tan stands for tangential direction, and rad stands for sagittal direction. At a cumulative probability of 97.7%, the lowest MTF value at a spatial frequency of 62 cycles/mm for the tolerances at the 1.00 field tan position is 0.3023, as shown in Figure 12. Except for the 0-degree field of view, the MTF curve is affected by astigmatism in other fields of view, and its tangential and sagittal MTF curves will be different. The maximum paraxial image height of 4.05 mm is defined as the 1.0 field of the collimating lens system. To ensure uniform image quality, the 1.0 field is divided into 11 evenly spaced points, corresponding to 0.0, 0.1, 0.2, …, up to 1.0 field. F1, F2, …, F11 are represented by tangential fields of view of 0.0, 0.1, 0.2, …, 1.0, respectively; and F12, F13, …, F21 are represented by radial fields of view of 0.1, 0.2, …, 1.0, respectively. In the tolerance analysis, we analyze each field of view, including tangential and radial values. We perform tolerance analysis on F1 to F21 in various directions across different fields of view, using different colors to represent each.

The values of the design MTF and design plus tolerance MTF for both the tangential and radial directions at a spatial frequency of 62 cycles/mm across all fields are shown in Table 6.

## 4. Volume and Weight of 50-Degree FOV Collimating Lens

The FOV of the 50-degree collimating lens design features an aperture stop located on the first surface of the lens, with the entrance pupil diameter of 4 mm. Four plastic aspherical lenses are used to achieve better lens quality. Shortening the gaps between the plastic lenses and the center thickness of the plastic lenses results in a smaller lens volume, as shown in Figure 6 and Table 3. The collimating optical system is divided into two parts: the lens part and the image part. The lens part consists of the first four plastic lenses at the front of the collimating optical system; the image part includes the microdisplay protection glass (BSC1-HOYA glass, 0.7 mm thick) and the microdisplay emitting surface, which is the image surface. Regarding the lens, since the aperture stop is located on the first lens, the first lens has the smallest clear aperture. The gap between the second and first lenses is tiny, which can limit the clear aperture of the second lens. The second lens is a convex lens, which effectively reduces the clear aperture of the third lens. To reduce the clear aperture of the fourth lens, we increase the image space chief ray angle θ_CA_ to θ_CA_ = 30.786° (θ_CA_ > 25°) and reduce the exit pupil diameter D_ex_ to 3.131 mm, as shown in Table 7.

**Figure 13:**
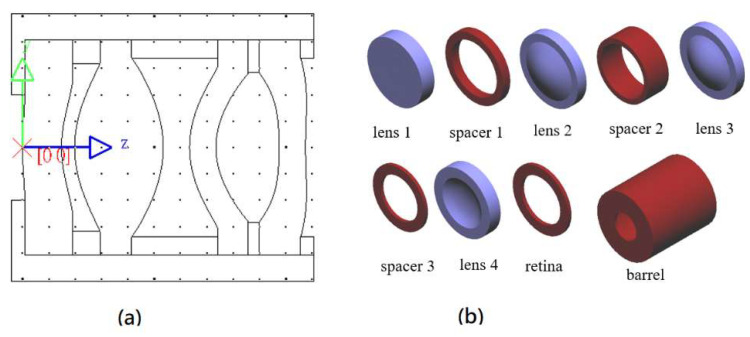
Assembly structure diagram of the internal optical and mechanical components of the lens. (**a**) a perspective view of the assembled lens; (**b**) internal components of the lens include four lenses, three spacers, one retina, and barrel.

The FOV of the 50-degree collimating lens design has a maximum clear aperture of 6.798 mm on the second surface of the second lens. To facilitate lens fixation and clamping, the lens diameter after manufacturing is generally called the finished aperture, which is larger than the clear aperture. In accordance with the requirements of the lens manufacturer, the finished aperture is 1.2 times the clear aperture, so the maximum finished aperture of the lens is 8.156 mm. To facilitate lens assembly, the finished aperture of all four lenses is 8.156 mm. The assembly structure of the optical and mechanical components inside the lens is shown in Figure 13. Figure 13a is a perspective view of the assembled lens, the optical components are arranged from left to right as lens 1, spacer 1, lens 2, spacer 2, lens 3, spacer 3, lens 4 and retina. Figure 13b shows that the internal components of the lens include four lenses, three spacers, one retina, and barrel.

**Figure 14:**
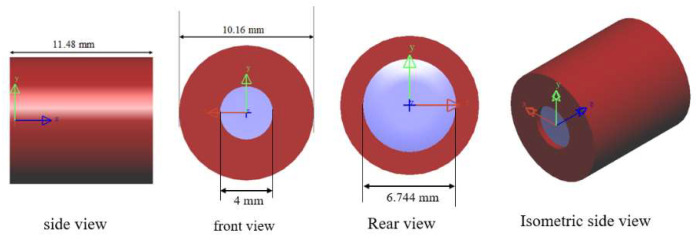
3D diagram and dimensions of the lens.

Table 8 shows the volume and weight of the various optical and mechanical components within the lens. The barrel, made of PMMA plastic, serves as the lens housing. It has a mass of 0.4132 g, an outer diameter of 10.156 mm, and a length of 11.48 mm. The inner ring is hollow with an inner diameter of 8.156 mm. The barrel ring’s thickness is fixed at 1 mm. The three-piece spacer structure is a mechanical component in the form of a ring that fixes the two lenses. It is made of an aluminum magnesium alloy. The retina is the ring structure that locks the final lens in place. It is made of PMMA plastic. The four lenses, three spacers, and one retina are finally assembled into the barrel, resulting in a total mass of 1.089 g. Figure 14 shows the 3D image and dimensions of the lens. The lens is a cylinder with a radius of 5.08 mm and a length of 11.48 mm; the resulting lens volume is 0.931 cm^3^.

As mentioned above, the 50-degree FOV collimator is designed with four aspherical lenses to balance optical quality and lens length. To reduce the size of the lens, we take the following steps:

Step 1: Select a smaller microdisplay (0.32 in).

Step 2: Set a smaller entrance pupil diameter (D_en_ = 4 mm) under a fixed F-number (F-number = 2.17) and smaller lens volume.

Step 3: Use the minimum number of lenses (4 pieces).

Step 4: Ensure that the thickness of the lenses and the gap between them is not too large.

Step 5: Place the aperture stop on the first surface of the lens to obtain the minimum clear aperture of the first lens.

Step 6: Arrange the focal length of four plastic lens symbols in the combination −, +, −, −, to reduce the clear aperture of the third lens.

Step 7: Set a smaller exit pupil diameter (D_EX_ = 3.131 mm).

Step 8: The image space chief ray angle (θ_CA_) is larger (θ_CA_ = 30.786°), which reduces the clear aperture of the fourth lens.

The data in brackets above relates to the FOV of the 50° collimating lens design in this article.

Table 9 compares the performance of collimating lens systems in related papers on AR glasses. This paper achieves several research results: minimum optical distortion of <0.21%; the best MTF performance of >0.23 at 250 cycles/mm; the lightest lens mass of 1.09 g; and the smallest volume of 0.93 cm^3^.

## 5. Conclusions

This paper presents a compact, wide field-of-view (50°) collimating lens system designed specifically for lightguide-based augmented reality (AR) glasses. The main purpose of collimating lens design is lightweight, and the lightweight of the lens is related to the size of the microdisplay, the effective aperture of the lens, the position and size of the entrance pupil, the position and size of the exit pupil, the number of lenses, the thickness and gap between the lenses, and the arrangement of the lens symbols. First, we selected 0.32-inch microdisplays, which yielded a minimum image height of 4.05 mm. The effective aperture of the lens is related to five reference rays. Five reference rays were defined, and then the half FOV (25°) was evenly divided into 11 fields of view. Five reference rays were traced for each field of view, for a total of 55 reference rays. The effective aperture of each lens surface (including the stop and image plane) was calculated. Rays were traced along each lens surface using these 55 rays. The effective aperture of each surface was the highest ray height among these 55 rays. The optimal pupil diameter for the human eye is 4 mm, so we chose a collimator with a 4 mm entrance pupil for our design. Since the entrance pupil is located on the first lens surface, the minimum effective aperture of the first lens element is 4 mm. The exit pupil position determines the image-side chief ray angle. The effective aperture of the last lens surface is inversely proportional to the image-side chief ray angle and directly proportional to the exit pupil diameter and the image plane height. Finally, the design used four plastic aspherical lenses with a maximum lens thickness or inter-lens gap of 3.368 mm. The focal length of the four plastic lens symbols (−, +, −, −) was arranged to reduce the clear aperture of the third lens. The resulting lens weighed only 1.09 g and had a volume of 0.93 cm^3^. Compared to a 2.5 g US penny, the weight of the proposed lens is only 43.6% of that, validating the feasibility of this design approach. Furthermore, compared to existing journal articles on collimating lens design for AR glasses, this lens design also achieved the lowest weight and volume.

This work builds upon previous research findings and proposes a compact design for collimating lenses for AR glasses, thereby reducing their size and weight for wearable applications. Consequently, we present a novel optical design for a compact collimating lens system designed for lightguide-based augmented reality (AR) glasses, achieving a wide field of view (FOV) of 50° using a minimal four-element aspheric plastic lens configuration. This design meets the stringent requirements of emerging wearable displays, striking a balance between high imaging performance, compactness, lightweight structure, and manufacturability.

## Figures and Tables

**Table 1:** Microdisplay specifications.

Parameters	Specification
Pixel number	800 × 600
Pixel size	8.1 μm × 8.1 μm
Effective area	6.48 mm × 4.86 mm
Diagonal length of the display	8.1 mm (0.32 inch)

**Table 2:** The specifications of the collimating lens.

Parameters	Specifications
Entrance pupil	4 mm
Diagonal FOV	50°
Horizontal FOV	40.94°
Vertical FOV	31.28°
Lens clear aperture maximum	≤6.8 mm
Focal length	8.68 mm
F-number	2.17
Image height	4.05 mm
Protective glass material	BSC1(HOYA)
Protective glass thickness	0.7 mm
MTF (62 cycles/mm)	≥0.7
Angular resolution (pixel per degree)	20 PPD
Angular magnification	28.802

**Table 3:** Lens design data of the collimating lens.

Surface Number	Surface Type	Radius (mm)	Thickness (mm)	Glass	Clear Aperture (mm)
Object	Sphere	Infinity	Infinity		
Stop	Sphere	Infinity	0		4.000
2	Asphere	10.6751	1.483	‘OKP-1_30’	4.083
3	Asphere	4.4966	0.500		5.085
4	Asphere	4.3777	3.368	PMMA_SPECAL	6.378
5	Asphere	−4.3101	1.000		6.798
6	Asphere	3.8496	1.000	‘OKP-1_30’	6.145
7	Asphere	2.9794	2.400		5.604
8	Asphere	−13.7379	1.000	‘OKP-1_30’	5.719
9	Asphere	15.2466	0.700		6.774
10	Sphere	Infinity	0.700	BSC1_HOYA	7.438
11	Sphere	Infinity	0.700		7.776
12	Image	Infinity	0.000		8.307

**Table 4:** Aspherical surface data for 50-degree FOV collimating lens.

Surface Number	K	A	B	C	D
**2**	−120	0.335966 × 10^−2^	−0.213406 × 10^−2^	0.302968 × 10^−3^	−0.192055 × 10^−4^
**3**	−5.161698	−0.148158 × 10^−2^	−0.606542 × 10^−3^	0.717905 × 10^−4^	−0.338550 × 10^−5^
**4**	−0.800282	−0.149620 × 10^−2^	−0.182126 × 10^−3^	0.211560 × 10^−4^	−0.100896 × 10^−5^
**5**	−4.456061	−0.298062 × 10^−2^	0.193746 × 10^−3^	−0.382837 × 10^−5^	−0.339511 × 10^−6^
**6**	−0.966918	−0.721381 × 10^−3^	−0.108456 × 10^−3^	0.995474 × 10^−5^	−0.119197 × 10^−5^
**7**	−2.644408	0.454588 × 10^−2^	−0.288402 × 10^−3^	0.113949 × 10^−4^	0.201825 × 10^−6^
**8**	−74.465683	−0.133534 × 10^−1^	0.113535 × 10^−2^	−0.135322 × 10^−3^	0.838188 × 10^−5^
**9**	−15.581008	−0.702624 × 10^−2^	0.481560 × 10^−3^	−0.267416 × 10^−4^	0.797422 × 10^−6^

**Table 5:** Tolerance parameter range.

Type	Minimum	Maximum	Increment
**DLF (fringe)**	1	5	0.5
**DLT (mm)**	0.003	0.02	0.005
**DLN**	0.0001	0.0005	0.0001
**DLV**	0.001	0.005	0.001
**CYD (fringe)**	1	1.5	0.1
**CYN (fringe)**	1	1.5	0.1
**TRX (arcmin)**	1	3	0.1
**TRY (arcmin)**	1	3	0.1
**BTY (arcmin)**	1	3	0.1
**BTX (arcmin)**	1	3	0.1
**DSX (mm)**	0.002	0.01	0.001
**DSY (mm)**	0.002	0.01	0.001

**Table 6:** Design and tolerance MTF at spatial frequency 62 cycles/mm.

Field	Spatial Frequency (cycles/mm)	Azimuth	Design MTF	Design Plus Tolerance MTF
**0**	62	Tangential	0.8726	0.8193
**0.1**	62	Tangential	0.8660	0.8293
**0.2**	62	Tangential	0.8490	0.7863
**0.3**	62	Tangential	0.8438	0.7760
**0.4**	62	Tangential	0.8453	0.7801
**0.5**	62	Tangential	0.8333	0.7410
**0.6**	62	Tangential	0.8166	0.7061
**0.7**	62	Tangential	0.7860	0.6409
**0.8**	62	Tangential	0.7495	0.5461
**0.9**	62	Tangential	0.7436	0.4959
**1.0**	62	Tangential	0.6917	0.3023
**0.1**	62	Radial	0.8683	0.8220
**0.2**	62	Radial	0.8645	0.8212
**0.3**	62	Radial	0.8644	0.8223
**0.4**	62	Radial	0.8661	0.8205
**0.5**	62	Radial	0.8545	0.7996
**0.6**	62	Radial	0.8458	0.7804
**0.7**	62	Radial	0.8337	0.7616
**0.8**	62	Radial	0.8156	0.7341
**0.9**	62	Radial	0.7745	0.6793
**1.0**	62	Radial	0.7228	0.6173

**Table 7:** Parameters for calculating exit pupil position and size.

**δ**	**−2.408 mm**	**h′**	**4.050 mm**
**δ′**	−7.401 mm	EFL	8.680 mm
**S**	2.408 mm	BFL	1.279 mm
**S′**	1.855 mm	θ_CA_	30.786°
**S + δ**	0 mm	M_T_	0.7828
**S′ + δ′**	−5.516 mm	D_ex_	3.131 mm

**Table 8:** Volume and weight of the internal optical and mechanical components of the lens.

Element Name	Material	Specific Gravity (g/cm^3^)	Volume (cm^3^)	Weight (g)
lens 1	OKP-1	1.22	0.1008	0.1230
spacer 1	Aluminum magnesium alloy	1.81	0.0210	0.0568
lens 2	PMMA	1.18	0.1053	0.1242
spacer 1	Aluminum magnesium alloy	1.81	0.0522	0.1410
lens 3	OKP-1	1.22	0.0907	0.1106
spacer 1	Aluminum magnesium alloy	1.81	0.0113	0.0305
lens 4	OKP-1	1.22	0.0659	0.0805
retina	PMMA	1.18	0.0079	0.0094
barrel	PMMA	1.18	0.03502	0.4132
Total				1.0892

**Table 9:** Comparison of collimating lens system performance in related AR glasses papers.

Author /Year	FOV (Degree)	EPD (mm)	F-Number	Lens Number	Microdisplay Size/Resolution	MTF	Distortion (%)	Weight (g)	Volume (cm^3^)
Hua [7]/2003	52.4	12	2.92	4	1.35 in 640 × 480	>0.2 at 30 cycles/mm	< 2.5%	8	3.53
Hua [8] /2007	56	10	3.2	4	1.3 in 640 × 480	>0.3 at 20 cycles/mm	<3.8%	6	3.15
Zhang [9]/2008	55	10	2.16	5	0.88 in 1280 × 1024	>0.4 at 37 cycles/mm	<4.0%	8.2	2.66
Pan [10]/2015	30	8	3.66	4	0.61 in 800 × 600	>0.4 at 30 cycles/mm	<2.7%	NR	12.45
Tsai [11]/2017	60	6	2.57	8	0.7 in 1920 × 1080	>0.29 at 62 cycles/mm	<5.0%	NR	9.76
Sun [14]/2024	30	14	1.17	9	0.35 in 1280 × 720	>0.44 at 35 cycles/mm	<2.0%	NR	25.02
Sun [15]/2025	65	10	2.05	7	1.03 in 2560 × 2560	>0.50 at 60 cycles/mm	<0.82%	NR	22.61
This work	50	4	2.17	4	0.32 in 800 × 600	>0.23 at 250 cycles/mm	<0.21%	1.08	0.93

## Data Availability

The original contributions presented in this study are included in the article. Further inquiries can be directed to the corresponding author.
